# Efficient Deconvolution Architecture for Heterogeneous Systems-on-Chip

**DOI:** 10.3390/jimaging6090085

**Published:** 2020-08-25

**Authors:** Stefania Perri, Cristian Sestito, Fanny Spagnolo, Pasquale Corsonello

**Affiliations:** 1 Department of Mechanical, Energy and Management Engineering, University of Calabria, 87036 Rende, Italy; 2 Department of Informatics, Modeling, Electronics and System Engineering, University of Calabria, 87036 Rende, Italy; cristian.sestito@unical.it (C.S.); f.spagnolo@dimes.unical.it (F.S.); p.corsonello@unical.it (P.C.)

**Keywords:** image deconvolution, generative adversarial networks (GANs), field-programmable gate array (FPGA), heterogeneous embedded systems

## Abstract

Today, convolutional and deconvolutional neural network models are exceptionally popular thanks to the impressive accuracies they have been proven in several computer-vision applications. To speed up the overall tasks of these neural networks, purpose-designed accelerators are highly desirable. Unfortunately, the high computational complexity and the huge memory demand make the design of efficient hardware architectures, as well as their deployment in resource- and power-constrained embedded systems, still quite challenging. This paper presents a novel purpose-designed hardware accelerator to perform 2D deconvolutions. The proposed structure applies a hardware-oriented computational approach that overcomes the issues of traditional deconvolution methods, and it is suitable for being implemented within any virtually system-on-chip based on field-programmable gate array devices. In fact, the novel accelerator is simply scalable to comply with resources available within both high- and low-end devices by adequately scaling the adopted parallelism. As an example, when exploited to accelerate the Deep Convolutional Generative Adversarial Network model, the novel accelerator, running as a standalone unit implemented within the Xilinx Zynq XC7Z020 System-on-Chip (SoC) device, performs up to 72 GOPs. Moreover, it dissipates less than 500 mW@200 MHz and occupies ~5.6%, ~4.1%, ~17%, and ~96%, respectively, of the look-up tables, flip-flops, random access memory, and digital signal processors available on-chip. When accommodated within the same device, the whole embedded system equipped with the novel accelerator performs up to 54 GOPs and dissipates less than 1.8 W@150 MHz. Thanks to the increased parallelism exploitable, more than 900 GOPs can be executed when the high-end Virtex-7 XC7VX690T device is used as the implementation platform. Moreover, in comparison with state-of-the-art competitors implemented within the Zynq XC7Z045 device, the system proposed here reaches a computational capability up to ~20% higher, and saves more than 60% and 80% of power consumption and logic resources requirement, respectively, using ~5.7× fewer on-chip memory resources.

## 1. Introduction

In the last few years, both Convolutional and Deconvolutional Neural Networks (CNNs and DCNNs) have been extensively used in deep-learning applications, such as object generation [1], image segmentation [2] and high-resolution imaging [3]. In such a scenario, while deconvolutions aim at extrapolating new features from inputs, to furnish upsampled outputs, convolutions compact the most relevant features through a downsampling process. Despite this difference, the operations are performed in a similar way. Deconvolutions, in fact, can be thought as convolutions executed on padded and strided inputs [4]. It represents the backbone of segmentation and super-resolution algorithms and it constitutes the basis of generative neural networks, successfully adopted to: synthesize realistic photographs or cartoons; perform images translation tasks; predict future frames in video sequences.

In typical deep-learning applications, owing to the huge amount of Multiply Accumulations (MACs) needed to perform convolutions and deconvolutions, the overall computational complexity can become an issue, especially when operating in real time is mandatory [5]. The bottleneck introduced by these operations is even more emphasized when software-based designs are implemented by Central Processing Units (CPUs), which provide limited parallelism. Conversely, as is well known, the use of Graphics Processing Units (GPUs) certainly can alleviate performance issues, but, unfortunately, they are not suitable for energy-constrained environments. Indeed, the latter can take advantages from Application-Specific Integrated Circuits (ASICs) and Field-Programmable Gate Arrays (FPGAs) that are widely recognized as appropriate hardware platforms to trade-off performance and power efficiency [6]. As a further advantage, FPGA-based designs also ensure flexibility and low costs to be achieved. However, although, as exhaustively reviewed in [7], plenty of FPGA-based accelerators can be found in the literature for CNNs, existing works focusing on the design of FPGA-based engines suitable for hardware-accelerating DCNNs are still few [8,9,10,11,12,13,14,15,16,17,18] and this problem is still open.

This paper presents a novel purpose-designed custom hardware accelerator to perform deconvolutions efficiently. The proposed Deconvolution Layer Processing Element (DLPE) was designed with embedded capability, to be easily integrated within virtually any heterogeneous FPGA-based System-on-Chip (SoC). Such a design approach was selected since it is effective in boosting performance and trading off power consumption and costs [19]. In fact, it merges the powerfulness of a dedicated Processing System (PS), suitable for control and non-time critical tasks, and the flexibility of a Programmable Logic (PL) fabric that can host hardware accelerators purposely designed for computationally intensive operations, such as convolutions and deconvolutions.

The FPGA-based hardware structure proposed here to speed up deconvolutions has been designed with a wide variety of applications in mind. Therefore, the main objectives have been: (1) achieving high-speed performance; (2) limiting the hardware resources requirements and the power consumption; and (3) making the accelerator easily scalable to comply with resources available within both high- and low-end FPGA-based SoCs. As demonstrated in the following, the novel accelerator satisfies all the above goals not only by smartly using logic and routing resources, but also by adopting an efficient data transfer policy to read and write data from/to an external memory. The proposed design complies with the Advanced eXtensible Interface (AXI4) protocol [20] and, therefore, it can be easily integrated within modern heterogeneous embedded systems.

As a case study, the novel engine has been employed to accelerate Deep Convolutional Generative Adversarial Networks (DCGANs) [21]. Such a kind of applications could certainly benefit from the latest advanced highly integrated hardware/software platforms, such as the Xilinx’s Versal™ Adaptive Compute Acceleration Platform (ACAP), very recently presented in [22]. However, for purposes of comparison with state-of-art competitors, the Xilinx Zynq XC7Z020 (Xilinx, San Jose, CA, USA), XC7Z045 (Xilinx), XC7Z100 (Xilinx) [23] and the Virtex-7 XC7VX690T (Xilinx) [24] devices have been used as the implementation platforms to characterize the proposed accelerator when running either as a Standalone Unit (SU) or as a part of an Embedded System (ES). When compared to the designs presented in [9,11,15,16,17,18], both the SU and the ES implementations proposed here exhibit remarkably higher throughput and they employ significantly lower amounts of Look-Up Tables (LUTs), Flip-Flops (FFs), on-chip Blocks of Random Access Memory (BRAMs), and Digital Signal Processors (DSPs). As an example, when implemented within the XC7Z020 device, the proposed ES is 50% faster than [9], performs ~20.7× more GOPs, and it occupies 49.8%, 42.7%, 36.6% and 5% less LUTs, FFs, BRAMs, and DSPs, respectively, dissipating just 1.73 W@150 MHz.

Ultimately, the main contributions and novelties provided in this work can be summarized as follows:

- an easily scalable engine for deconvolution is proposed that can be fruitfully employed within different CNNs/DCNNs models;
- the novel architecture exploits both data- and circuit-level parallelism, thus it is suitable for accelerating deconvolutions within both high- and low-end FPGA-based SoCs;
- the on-chip DSPs resources are fully exploited to perform both multiplication and addition operations, therefore speed performance higher than the state-of-the art competitors is achieved with reduced logic resources requirements and power consumption;
- the proposed hardware accelerator complies with the Advanced eXtensible Interface (AXI4) protocol [20] and, therefore, it can be easily integrated within modern heterogeneous embedded systems;
- input and output data streams are read and written from/to an external memory through the raster-order transfer policy; the latter allows data packets to be moved concurrently, thus improving the global latency.

The rest of the paper is organized as follows: Section 2 provides a background on deconvolution algorithms and related works concerning FPGA-based accelerators; Section 3 describes the architecture of the proposed hardware accelerator; tests and results are discussed in Section 4; finally, in Section 5, conclusions are drawn.

## 2. Background, Related Works, and Motivations

Representative examples of CNN and DCNN models requiring deconvolutions are provided by the Fully Convolutional Network (FCN) described in [25], the U-Net architecture presented in [26], the Super-Resolution CNN (SRCNN) discussed in [3], and the generative DCNNs demonstrated in [21]. By examining those network models, it can be easily observed that although a CNN consists of cascaded convolutional layers (CONVs), a DCNN comprises a certain number of cascaded deconvolutional layers (DECONVs). In both cases, cascaded layers are interleaved by optional stages, such as non-linearity, normalization, pooling [5] and unpooling [27]. To meet a precise task, such as classification and segmentation, along the whole architecture of a CNN, each CONV extracts even more abstract features from 2D input feature maps (*ifmaps*). Moreover, different bottom layers concur to dictate the target of each model. As an example, while classification networks adopt fully connected layers to arrange extracted features in categories, segmented images are built by means of up-sampling stages, including DECONVs [28]. This means that performing deconvolutions efficiently provides benefits not only to DCNNs, but also to a certain class of CNNs. 

Generally speaking, a DECONV layer receives a set of *N_C_* 2D *H × W ifmaps* and produces *N_F_* 2D *Ho × Wo* output feature maps (*ofmaps*). To do this, *N_F_* sets of *N_C_* 2D *K × K* filters kernels are used. More precisely, each *ifmap* is filtered by using its own kernel and the *N_C_* results obtained in this way are combined by means of a pixel-wise addition, thus providing an *ofmap*. This mechanism is repeated for all the *N_F_* sets of filters. Ultimately, it can be said that at the top-level, DECONVs act quite similarly to CONVs. However, when the bare deconvolution and convolution operations are examined, significant differences become more or less evident, depending on the computational strategy adopted within DECONVs. 

The method adopted in [4] computes deconvolutions by executing typical convolutions on zero-padded and strided *ifmaps*. More exactly, with *K*, *S*, and *P* being the kernel size, the stride, and the padding, respectively, the deconvolution result is obtained by interleaving *S* − 1 zeros between each pair of consecutive input pixels and then performing the classical convolution operation adopting the kernel size *K*’ = *K*, the stride *S*’ = 1, and the padding *P*’ = *K* − *P* − 1. As an example, such a strategy is exploited in the FlexiGAN framework presented in [8] to generate accelerators for Generative Adversarial Networks (GANs). Unfortunately, this approach requires data and filter be properly reorganized thus making additional control logic necessary and severely limiting the achievable overall performance. As a further drawback, the above-described zero-padding and striding strategies lead to unbalanced workloads since they introduce useless zeroed MAC operations. 

The efficient design strategy recently presented in [17] overcomes the above issues by performing a kernel conversion to calculate all the pre-addable weight combinations. The output of this process is a new set of filters that can be directly applied to the *ifmaps* to perform a traditional 3D convolution. Such a strategy allows drastically reducing the computational complexity and introduces remarkable speed-up either over other FPGA accelerators or over GPU platforms. 

A completely different technique has been recently proposed in [9]. Such an approach multiplies each input pixel by the relative *K × K* deconvolution kernel, thus furnishing a block of *K × K* output products. It is worth noting that neighboring input pixels lead to overlapping output blocks. With *S* being the supported stride, up to *K* − *S* overlapping rows and columns must be properly managed to provide the correct deconvolution result. Unfortunately, the Deconvolution Engine (DE) proposed in [9] does not manage overlaps efficiently. In fact, to recognize no-overlapping blocks, it applies the reverse looping that requires the computation of input coordinates at each filtering step, with obvious penalties in terms of computational complexity and delay. 

Appreciable improvements were introduced in [10,11,12,13,14,15,16,18]. The accelerator proposed in [10] was purposely designed to accomplish the semantic segmentation. It uses separate convolution and deconvolution engines, but, due to its hardware resources requirements, it is not easily exploitable within low-end FPGA-based SoCs. Moreover, while multiplications are performed as fast as possible, by exclusively exploiting DSP slices, the additions required to proper manage overlapping rows/columns are performed through configurable logic resources, thus severely limiting the achievable performance. As a further drawback is that the accelerator does not support the final cropping. Therefore, as demonstrated in [11], auxiliary logic modules must be introduced to crop pixels on the borders around the *ofmaps*. 

The above-described structure was further improved in [12], where a unique engine is used to perform both convolutions and deconvolutions to meet the remote segmentation task. Such an accelerator employs just one MAC unit that operates in a serial manner, whereas it exploits a high level of parallelism at both *fmaps* and filters levels. Unfortunately, due to its high hardware resources requirements, this accelerator can be actually exploited only within high-end FPGA-based SoCs. Moreover, it is not the most attractive solution for achieving the highest speed. In fact, it requires up to 4 clock cycles to furnish each deconvolved pixel, depending on how many overlapping pixels must be managed [12]. 

The innovative solutions proposed in [13,14] avoid the use of dedicated deconvolution engines by transforming deconvolutional layers into convolutional ones. In such a case, *N_F_ × S ofmaps* are provided instead of just *N_F_*, thus making a very high parallelism level necessary to achieve reasonable speed performance. Consequently, once again, the resulting hardware resources requirement makes the use of a high-end FPGA device necessary.

Although all the previous works faced the acceleration of 2D deconvolutions, the solution proposed in [15] is suitable also for 3D scenarios. It exploits the sparsity of input activations and weights to reduce the number of useless multiplications, introducing a compression scheme that further enhances the efficiency of the computational unit. However, the approach presented in [15] requires non-zero weights to be encoded in coordinate format. This task involves the calculation of the output coordinates of each deconvolved pixel, thus preventing the integration of such an accelerator in streaming-based heterogeneous embedded systems. 

As demonstrated in [16,18], also the well-known Winograd algorithm can be exploited to deploy deconvolutional layers of GANs on FPGAs efficiently. Indeed, the Winograd algorithm transforms 2D convolutions into element-wise multiplications implemented by simple additions and shifts operations. Despite the impressive speed performance achieved in [16,18] also thanks to the high parallelism adopted, this computational method introduces area and power overheads due to the pre- and post-processing operations required to transform feature maps and filters in the Winograd domain. 

## 3. The Proposed Hardware Accelerator

The approach adopted here to deconvolve one *H × W ifmap* by a *K × K* kernel with stride *S* consists of four main steps. In the first one, the generic input pixel *I*(*i*,*j*) is multiplied by the kernel and the resulting block of *K × K* products is properly arranged within the *ofmap* by occupying the *K × K* area starting at the position (*I × S, j × S*). As expected, neighboring resulting blocks, obtained from neighboring input pixels, have up to *K–S* overlapping rows/columns, which are summed up in the second step. Then, the above steps are repeated for all the input pixels. Finally, *P* pixels on the borders around the resulting *ofmap* are cropped, thus generating a *H*_0_ × *W*_0_ *ofmap*, with *H*_0_ and *W*_0_ being defined in Equation (1).

(1) $$H_{O}=S\times \left(H-1\right)+K-2P\;W_{O}=S\times \left(W-1\right)+K-2P$$

**Figure 1:**
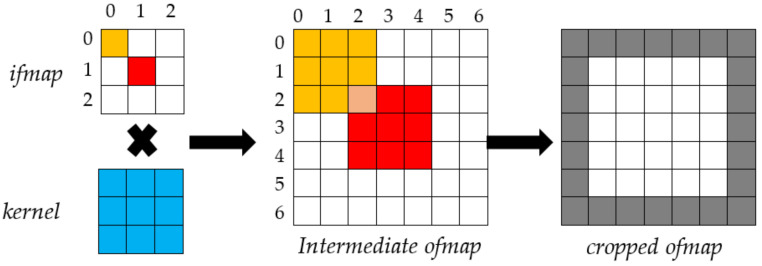
The adopted deconvolution approach.

The example depicted in Figure 1 shows how this approach deconvolves a 3 × 3 *ifmap* by using *K* = 3, *S* = 2 and *P* = 1. When deconvolved by the referred 3 × 3 kernel, the orange pixel *I*(0,0) in the *ifmap* leads to the orange 3 × 3 block of pixels starting at the location (0,0) in the intermediate *ofmap*. Similarly, by deconvolving the red pixel *I*(1,1) in the *ifmap*, the 3 × 3 red block starting at the location (2,2) in the intermediate *ofmap* is obtained. In addition, so on for all the other pixels. To complete the deconvolution correctly, the overlapping pixels within neighboring blocks in the *ofmap* are summed up. Finally, since *P* = 1, one pixel is cropped on the borders around the *ofmap*, as highlighted in grey. 

**Figure 2:**
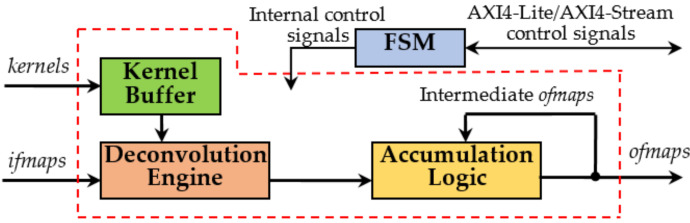
The top-level architecture of the Deconvolution Layer Processing Element. FSM: The Finite State Machine.

The top-level architecture of the novel accelerator, hereby named DLPE, is depicted in Figure 2. It can process *T_N_ ifmaps* (*if*_0_,…, *if*_*TN*−1_) and *T_M_* kernels in parallel, thus operating on *T_M_ ofmaps* (*of*_0_,…, *of*_*TM*−1_) at the same time. In particular, the DLPE can receive *P_N_* pixels from each *ifmap* and can furnish *P_M_* elements for each *ofmap* contemporaneously, with *P_M_ = S × S × P_N_*. Obviously, *T_N_*, *T_M_*, *P_N_, and P_M_* strictly depend on the resources availability within the specific device chosen as the realization platform. Anyway, when the number *N_C_* of *ifmaps* and/or the number *N_F_* of kernels to be processed are greater than *T_N_* and *T_M_*, respectively, the overall computation is completed within multiple steps, as discussed in the following. 

The top-level architecture of the novel accelerator, hereby named DLPE, is depicted in Figure 2. It can process *T_N_ ifmaps* (*if*_0_,…, *if*_*TN*−1_) and *T_M_* kernels in parallel, thus operating on *T_M_ ofmaps* (*of*_0_,…, *of*_*TM *−1_) at the same time. In particular, the DLPE can receive *P_N_* pixels from each *ifmap* and can furnish *P_M_* elements for each *ofmap* contemporaneously, with *P_M_ = S × S × P_N_*. Obviously, *T_N_*, *T_M_*, *P_N_, and P_M_* strictly depend on the resources availability within the specific device chosen as the realization platform. Anyway, when the number *N_C_* of *ifmaps* and/or the number *N_F_* of kernels to be processed are greater than *T_N_* and *T_M_*, respectively, the overall computation is completed within multiple steps, as discussed in the following. 

The novel accelerator has been designed supposing that both *ifmaps* and kernels are stored within an external Double Data Rate (DDR) memory and, as shown later, they can be simply uploaded and streamed towards the DLPE by auxiliary circuitries, such as Direct Memory Access (DMA) and/or Video DMA (VDMA) modules. Although the *F*-bit pixels of the *ifmaps* are streamed-in directly to the DE, the *N*-bit kernels coefficients are preliminarily locally stored within the Kernel Buffer and then provided to the DE at the proper time. The Accumulation Logic (AL) exploits fast adder trees to accumulate provisional results produced at the various computational steps and collected within on-chip memory resources until the last step is performed and the final *ofmaps* are generated. The Finite State Machine (FSM) orchestrates all the operations and makes the whole accelerator AXI4 [20] compliant. In fact, it takes care of managing all the activities related to data transfers, including the AXI4-Stream transactions through which the packed kernels coefficients and the *ifmaps* are received.

**Figure 3:**
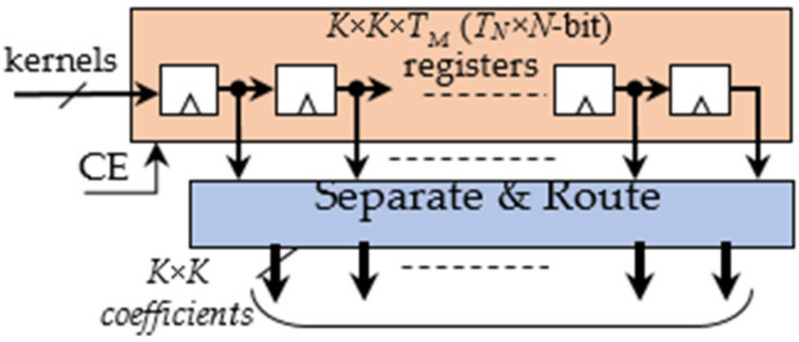
The Kernel Buffer.

**Figure 4:**
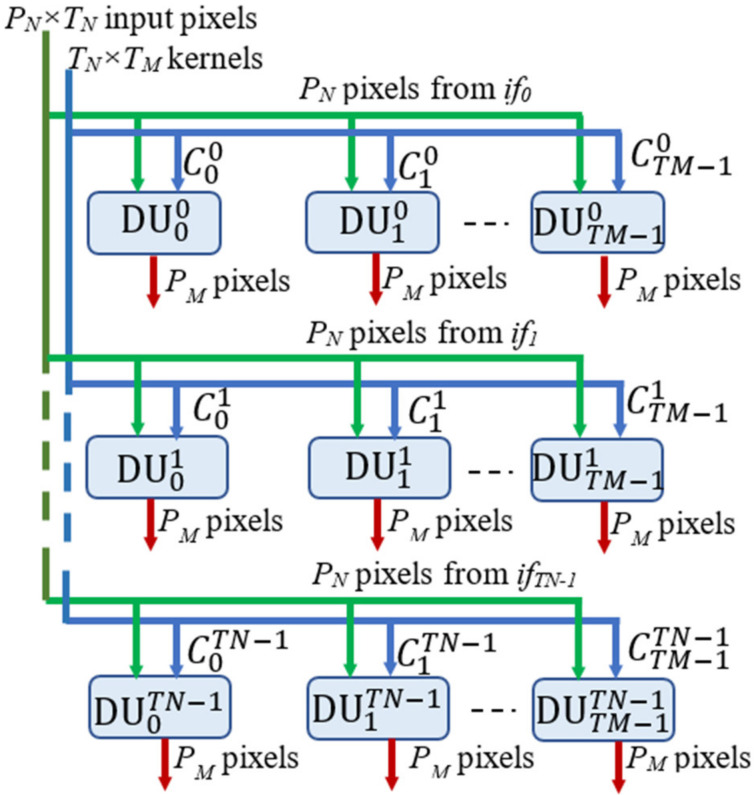
The architecture of the novel Deconvolution Engine.

The Kernel Buffer, shown in Figure 3, mainly consists of a register file able to store *K × K × T_M_ × T_N_* *N*-bit coefficients. At each clock cycle, the buffer receives the homologous coefficients related to the *T_N_ ifmaps if*_0_, …, *if*_*TN*−1_ and packed within one *T_N_ × N*-bit word. This strategy allows uploading all the kernel coefficients processed in parallel by the DE within just *K × K × T_M_* clock cycles. The Separate and Route logic properly dispatches the coefficients to the DE. The latter is the computational core of the proposed DLPE and, as illustrated in Figure 4, it consists of *T_M_ × T_N_* Deconvolution Units (DUs) operating in parallel. At each clock cycle, the generic ${\mathrm{DU}}_{out}^{in}$ receives *P_N_* adjacent input pixels *I*(*i,j*), *I*(*i,j* + 1), …, *I*(*i,j* + *PN* − 1) from the *ifmap if_in_*, with *in* = 0, …, *TN* − 1, and deconvolves them with the relative *K × K* kernel $C_{out}^{in}$ as required to compute the *ofmap of_out_*, with *out* = 0, …, *TM* – 1. The input pixels are multiplied in parallel by the coefficients of the kernel and *P_N_* blocks of *K* × *K* products are computed contemporaneously. 

**Figure 5:**
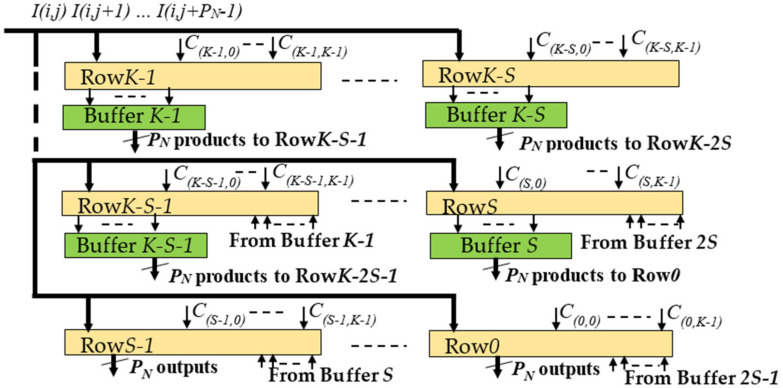
The structure of the generic DU.

Each DU was structured as depicted in Figure 5 to manage efficiently the overlapping rows/columns between these neighboring blocks of products. Figure 5 shows that the generic DU consists of the *K* modules Row*x*, with *x* = 0, …, *K* − 1, each using an appropriate number of DSPs, depending on the supported parallelism level. Furthermore, to guarantee the proper time alignment of the overlapping products, First-In-First-Out (FIFO) Buffers are exploited. 

**Figure 6:**
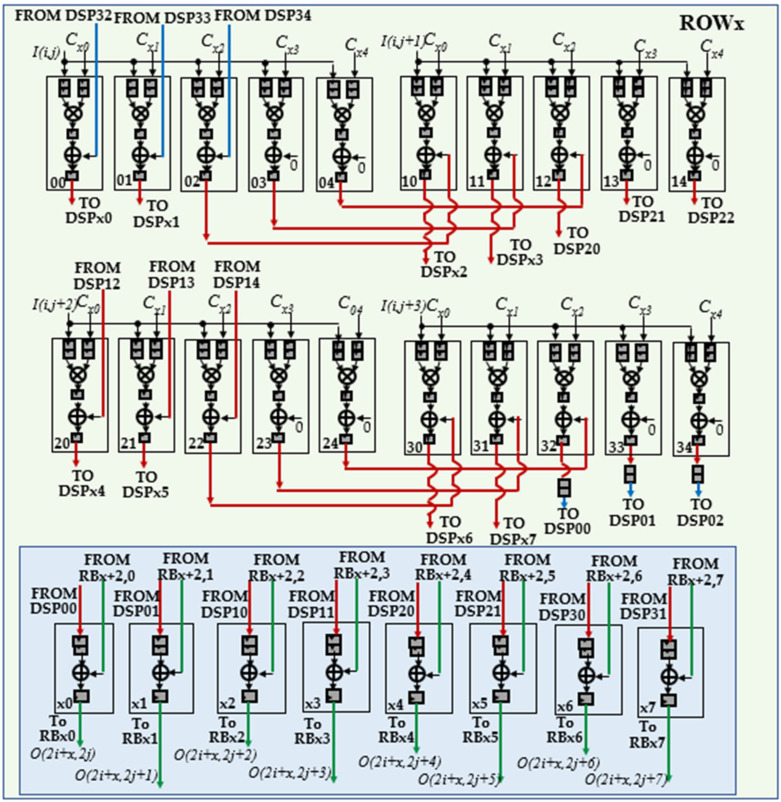
The architecture of the module Row*x* for *P_N_* = 4, *K* = 5, *S* = 2.

**Figure 7:**
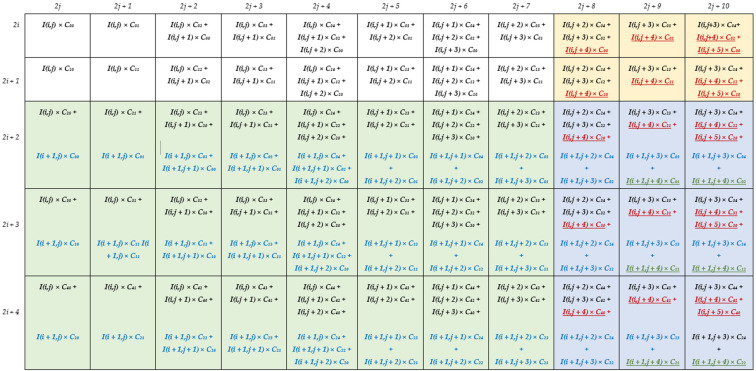
The operations performed by the generic DU when *P_N_* = 4, *K* = 5, *S* = 2. Black: operations performed at the current clock cycle; red: operations performed at the next clock cycle; blue: operations performed on the pixels belonging to the (*i* + 1)-th row and the (*j*),…(*j* + 3)-th columns; green: operations performed on the pixels belonging to the (*i* + 1)-th row and the (*j* + 4)-th column.

To better explain how the generic DU performs deconvolutions, let us consider, as an example, the kernel size *K* = 5, the stride *S* = 2 and *P_N_* = 4. In this case, *x* ranges from 0 to 4 and five modules Row*x* are required, each one, as reported in Figure 6, consisting of 20 DSPs. The latter are named *dy*, with *d* = 0, …, 3 and *y* ranging from 0 to 4, to indicate that they multiply the input pixel *I*(*i,j* + *d*) by the kernel coefficient *C*_(*x,y*)_. The additional DSPs *x*0, …, *x*7 are required only within the modules Row0, Row1, and Row2 to manage the overlapping rows. On the contrary, the *S* × *P_N_* results computed by Row3 and Row4 are directly provided by the DSPs 00, 01, 10, 11, 20, 21, 30, and 31. All the multiplications and the additions performed in the examined example by the generic DU are summarized in Figure 7 that also shows, for each entry, the related row and column indices within the intermediate *ofmap* currently computed. Since *K – S* = 3, as highlighted by colored entries, each block of products computed by the DU has three columns and three rows overlapped with neighboring blocks. 

To better explain how the generic DU performs deconvolutions, let us consider, as an example, the kernel size *K* = 5, the stride *S* = 2 and *P_N_* = 4. In this case, *x* ranges from 0 to 4 and five modules Row*x* are required, each one, as reported in Figure 6, consisting of 20 DSPs. The latter are named *dy*, with *d* = 0, …, 3 and *y* ranging from 0 to 4, to indicate that they multiply the input pixel *I*(*i,j* + *d*) by the kernel coefficient *C*_(*x,y*)_. The additional DSPs *x*0, …, *x*7 are required only within the modules Row0, Row1, and Row2 to manage the overlapping rows. On the contrary, the *S* × *P_N_* results computed by Row3 and Row4 are directly provided by the DSPs 00, 01, 10, 11, 20, 21, 30, and 31. All the multiplications and the additions performed in the examined example by the generic DU are summarized in Figure 7 that also shows, for each entry, the related row and column indices within the intermediate *ofmap* currently computed. Since *K – S* = 3, as highlighted by colored entries, each block of products computed by the DU has three columns and three rows overlapped with neighboring blocks. 

It is easy to observe that several kinds of overlapping products must be managed. The products related to the *P_N_* adjacent input pixels, currently received by the DU, have the row index equal to *i* and the column index ranging between *j* and *j* + 3. These products are reported in Figure 7 with black characters and their overlaps are managed through the red interconnections visible in Figure 6. Conversely, the products reported in Figure 7 with red characters are being computed at the next clock cycle, when the DU is receiving the next *P_N_* adjacent pixels, i.e., *I*(*i,j* + 4), …, *I*(*i,j* + 7), as input. The column overlaps related to these products are managed through the blue interconnections used in Figure 6 to transfer the delayed outputs produced by the DSPs 32, 33 and 34 towards the DSPs 00, 01, and 02, respectively. Finally, the products reported in Figure 7 with blue characters involve the pixels *I*(*i* + 1, *j*), …, *I*(*i* + 1, *j* + 3), which belong to the (*i* + 1)-th row of the *ifmap* currently processed. To receive these pixels as input, the DU must wait for all the pixels of the *i*-th row have been processed. As above illustrated in Figure 5, to guarantee the proper time alignment of these overlapping products, appropriate Buffers are exploited. In the examined example, they are required at the output of the three modules Row2, Row3, and Row4. These overlapping products are managed within the modules Row0, Row1 and Row2 through the DSPs *x*0, …, *x*7 and the green interconnections depicted in Figure 6. Thanks to the fully pipelined adopted architecture, after the initial latency, each DU furnishes *S × S × P_N_* deconvolved pixels at every clock cycle. These deconvolved pixels are reported in the white entries of Figure 7 as provided by the modules Row0 and Row1. 

Obviously, resources requirements, latency and throughput rate of the DE depend on the *ifmaps* size *H × W*, as well as on *K*, *S, T_N_, T_M_, and PN*. In the generic scenario, each DU needs [*K × K* + *S* × (*K − S*)] × *P_N_* DSP slices to perform multiplications and to sum the overlapping neighboring products that are time aligned through *S* × (*K – S*) × *P_N_* row buffers, each being $\frac{S\;\times \left(W\;-\;1\right)\;+\;K}{S\;\times \;P_{N}}-2$ depth. 

The novel DE has been designed taking into account also the treatment of border pixels. This is a key aspect, since it affects the data flow of the input streams. In fact, each of the *T_M_ × T_N_* DUs operating in parallel receives its own *ifmap* in the raster order. At the end of each row, the DE stops the incoming stream of pixels for $\frac{S\;\times \;\left(W\;-\;1\right)+K}{S\;\times \;P_{N}}-\frac{W}{P_{N}}$ clock cycles. During this time, the zero-padding is applied through a proper multiplexing logic directly controlled by the FSM that also manages the AXI4 protocol signals coherently with the desired wait. At the end of the current step, the DE provides $\frac{S\;\times \;\left(H\;-\;1\right)\;+\;K}{S}-H$ padding rows, before acquiring the next group of *ifmaps* to perform the subsequent computational step. 

**Figure 8:**
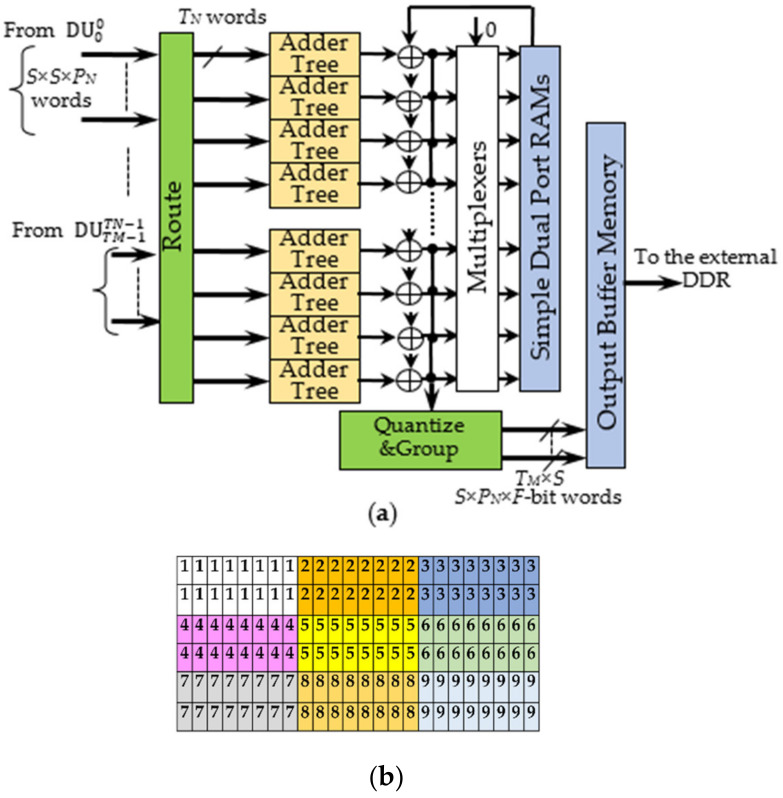
The Accumulation Logic: (**a**) the architecture; (**b**) the *ofmap* arrangement for *P_N_* = 4 and *S* = 2.

As above explained, each of the parallel DUs inside the DE outputs *T_M_* × *T_N_* blocks of *S × S × P_N_* deconvolved pixels. The homologous pixels within these blocks are accumulated to compose an intermediate *ofmap*. In turn, the intermediate *ofmaps* are accumulated step-by-step to each other until the DLPE provides the final result. The AL purposely designed to operate in this way is depicted in Figure 8a. The Route module receives blocks of deconvolved pixels from the DUs and sends them to *S × S × T_M_ × P_N_* adder trees taking into account that each group of *T_N_* homologous data must feed the same Adder Tree. The latter exploits DSPs to execute accumulations as fast as possible. The intermediate *ofmaps* provided by the adder trees are temporarily stored within local Simple Dual Port RAMs (SDPRAMs). They are resumed later to be accumulated with the intermediate *ofmaps* produced at the next step. During the last computational step, the final deconvolved pixels are generated. The Quantize and Group module quantizes the final deconvolved pixels to *F*-bit values and properly arranges them into packed words to be processed by the next layer, as required by the referenced DCNN (or CNN) model. Such packed words are stored within the Output Buffer Memory to be then sent towards the external DDR memory. In the meantime, the bank of Multiplexers visible in Figure 8a drives SDPRAMs with zeros input. In this way, the SDPRAMs are prepared for the next deconvolution task without wasting additional initialization time.

The adopted packing strategy takes into account that the DLPE produces *T_M_* × *T_N_* blocks of *S × S × P_N_* pixels per clock cycle. The example depicted in Figure 8b shows one 6 × 24 *ofmap* produced with *S* = 2 and *P_N_* = 4. Different colors are used to highlight the pixels furnished in parallel at a certain clock cycle so that, as an example, all the pixels located at the yellow entries are furnished at the 5th clock cycle. To ensure that the final *ofmap* is stored within the external DDR in the raster order, each block of pixels can be arranged in two words, each containing the pixels within the same row. Hence, in the example, the Quantize and Group module would furnish two *2 × P_N_* × *F*-bit words at every clock cycle. In the generic operating condition, this module packs the final deconvolved pixels within *T_M_* × *S* words, each being *S* × *P_N_* × *F*-bit wide. 

## 4. Implementation and Results

**Figure 9:**
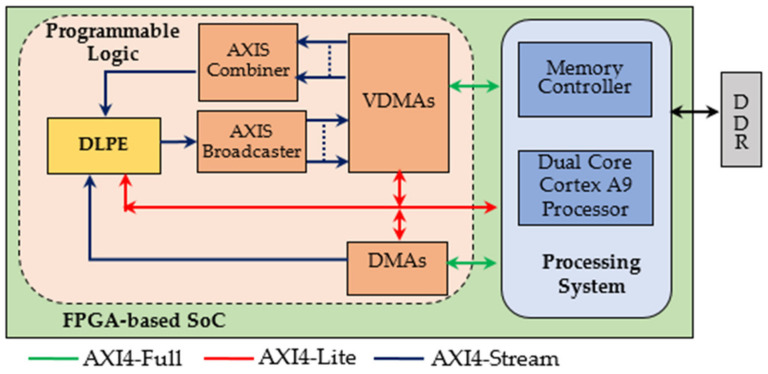
The referred embedded system architecture.

Custom designed parametric constructs were purposely written using the Very High-Speed Integrated Circuits Hardware Description Language (VHDL) to describe the proposed DLPE at the Register-Transfer-Level (RTL) abstraction. This approach allowed the novel hardware accelerator to be easily customized to different operating conditions and high computational speeds to be achieved by carefully using the available resources. The 2019.2 Vivado Design Suite has been used to perform simulations, synthesis, and implementations. For purposes of comparison with existing competitors, the DLPE described in the above Section has been exploited to accelerate the DCGAN neural network presented in [21]. In particular, the heterogeneous ES depicted in Figure 9 has been designed. Even though only implementations within Xilinx devices are detailed in the following, virtually any other devices family can be used for purposes of prototyping. In fact, the whole system mainly consists of the PS and the PL. As typically happens, the former is responsible for configuring the modules within the PL, for controlling the whole computation at the system level, and for performing non-time critical tasks. Conversely, the PL accommodates the novel DLPE and all the auxiliary circuitry required to manage the data transfers from/to the external DDR memory, as ruled by the AXI4 communication protocols. As detailed in the legend of Figure 9, different colors are used to distinguish connections supporting memory-mapped transactions from data streams.

The supported parallelism level is dictated by *T_M_*, *T_N_*, *P_M_*, and PN, which are properly set in accordance with the amount of resources available within the specific device chosen as the target implementation platform. As an example, using the low-end XC7Z020 Zynq device, with *T_M_* and *T_N_* being set to 2 and 3, respectively, *P_M_* = 4 and *P_N_* = 1 can be used. This means that $\left[\frac{N_{F}}{T_{M}}\right]$ pairs of *ofmaps* are computed, each within $[\frac{N_{C}}{T_{N}}]+1$ computational steps. Each module depicted in Figure 9 has its role: (1) the DMA [29] is responsible for uploading the kernels coefficients; (2) the VDMAs [30] are responsible for resuming and storing the *ifmaps* and the *ofmaps*; (3) the AXIS Combiner [31] synchronizes the parallel input data within a single data stream, then fed to the DLPE; (4) finally, the AXIS Broadcaster [31] separates the output pixels received in parallel from the DLPE depending on the *ofmap* they belong to. It is worth noting that the adopted data transfer policy allows the *ofmaps* to be directly arranged within the DDR memory in the raster order. Therefore, subsequent cascaded deconvolutional layers can process them, without requiring either complex management of the memory address space or expensive data reorganization. 

**Figure 10:**
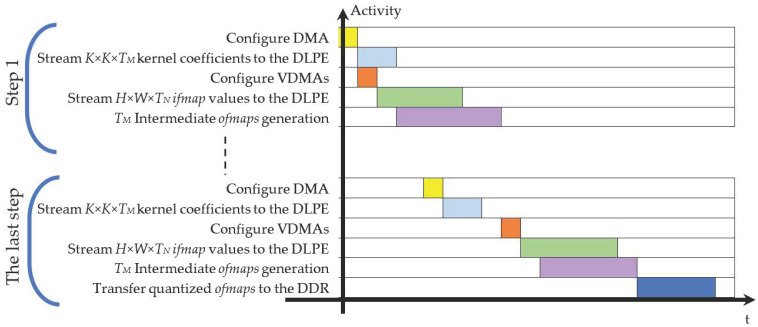
The computational flow of the architecture in Figure 9.

To better explain how the proposed DLPE is exploited in the ES of Figure 9, let examine the computational flow schematized in Figure 10. The latter details the main activities as performed over the time within multiple computational steps each providing *T_M_ ofmaps*. During the first step, the processor configures the DMA to specify which off-chip memory area must be accessed to read a block of *K* *× K × T_M_* kernel coefficients. These coefficients are then streamed towards the DLPE to be stored within the Kernel Buffer. In the meantime, the processor instructs the VDMAs to transfer *H* × *W ×* *T_N_ ifmap* values from the off-chip memory to the DLPE. After the initial latency, the latter will produce the intermediate *T_M_ ofmaps* that are on-chip stored for further accumulations. The above operations are repeated for all the subsequent steps, until the last one is executed. In this case, the VDMAs are also configured to transfer the final quantized *T_M_ ofmaps* from the DLPE to the external DDR memory. 

For purposes of comparison with state-of-the-art competitors, also designed to accelerate the DCGAN model presented in [21], several alternative implementations of the novel accelerator have been carried out and characterized using both low- and high-end devices. 

The obtained results are summarized in Table 1 in terms of: supported parallelism (*T_M_*, *T_N_*, *P_M_*, and *P_N_*), kernel size (*K*) and stride (*S*); resources requirements; running frequency; number of operations performed per second (GOPs); and, finally, dynamic power consumption. 

It is worth highlighting that while the designs presented in [15,16,17] are SUs, those demonstrated in [9,11,18] are embedded heterogeneous systems (ESs). For this reason, several SU and ES versions of the design here presented have been characterized and they are referenced in Table 1. The latter clearly shows that independently of the device used, the proposed implementations exhibit remarkable throughputs with reasonable resources requirements. Obviously, in comparison with the SU implementations, due to the auxiliary modules used to manage data transfers from/to the external DDR memory, the ES implementations occupy more LUTs, FFs, and on-chip BRAMs. Moreover, the PS obviously leads to an increased dynamic power consumption. 

From Table 1 it can be seen that the SU architectures presented in [15,17] exploit very high parallelism levels and operate with *K* = 3 and *S* = 1. Nevertheless, the design presented here, though it exploits a lower parallelism and operates with *K =* 5 and *S =* 2, which are more complex to manage than *K* = 3 and *S* = 1, at a parity of the device used, reduces the amount of occupied LUTs, FFs, and DSPs by ~86%, ~90% and ~43% with respect to [17]. Furthermore, it occupies 20 × less BRAMs and reaches a 1.5 × higher running frequency. Tests purposely performed on the novel accelerator have shown that when operating with *T_M_* = 2, *T_N_* = 4, *P_M_* = 16, *P_N_* = 4, *K* = 3 and *S* = 1, the resources requirements are further reduced and the consumed dynamic power is more than 45% lower than [17]. Analogously, when implemented within the XC7VX690T device, the proposed design saves a significant amount of occupied resources with respect to [15] that reaches a very high number of operations per second (GOPs) also thanks to the reduced kernel size and stride.

A further aspect to take into account is related to the high parallelism exploited in [15,16,17] at the *ifmaps* level (i.e., *T_N_*). Indeed, ad-hoc memory managements are necessary to allow either 64 or 128 homologous pixels belonging to as many *ifmaps* to be accessed contemporaneously. To support such irregular data access policies, the designs presented in [15,17] need a quite significant amount of on-chip BRAMs. As a drawback, this approach limits the scalability and the possibility of implementing these designs also within low-end devices, unless reducing the parallelism exploited, at the expense of the computational speed. Conversely, to keep data transfer to/from the external memory regular, as happens with the simple raster scan order, the novel accelerator mainly exploits pixel-level parallelism. This is a key feature to make the proposed design easily scalable and suitable for the implementation within low-end devices. Similar considerations arise for the accelerator demonstrated in [16]. However, the latter has the merit of supporting the 32-bit floating-point representation, which certainly leads to an overall quality higher than all the other solutions, but with a significant speed penalty. 

Among the compared ES implementations, as expected, that the one based on the reverse looping approach [9] is the slowest one. At the parity of the implementation device platform, in comparison with [9], the ES presented here occupies ~49.8% less LUTs, ~42.7% less FFs, 1.6 × less BRAMs and ~5% less DSPs. Moreover, it is ~20.7 × faster and achieves a density efficiency, evaluated as the ratio GOPs/DSPs, ~21.7 × higher. 

The proposed deconvolution architecture exhibits remarkable advantages also with respect to [11,18]. The significant reduction of occupied resources, achieved also with respect to these counterparts, is due to the more efficient architecture here exploited by the generic DU. In fact, the separate analysis, purposely performed varying *K* and *S*, demonstrated that the proposed DU always minimizes the amount of occupied LUTs and FFs. This happens because, in contrast with [11], DSPs are exploited to perform both multiplications and accumulations.

Among the ES implementations referenced in Table 1 as the state-of-the-art competitors, the design presented in [18] is certainly the most competitive in terms of speed performance. However, the novel ES exhibits a computational capability ~19.5% higher, it occupies ~12 × less LUTs, ~5.8 × less BRAMs and ~7% less DSPs, thus dissipating ~60% less power. 

**Figure 11:**
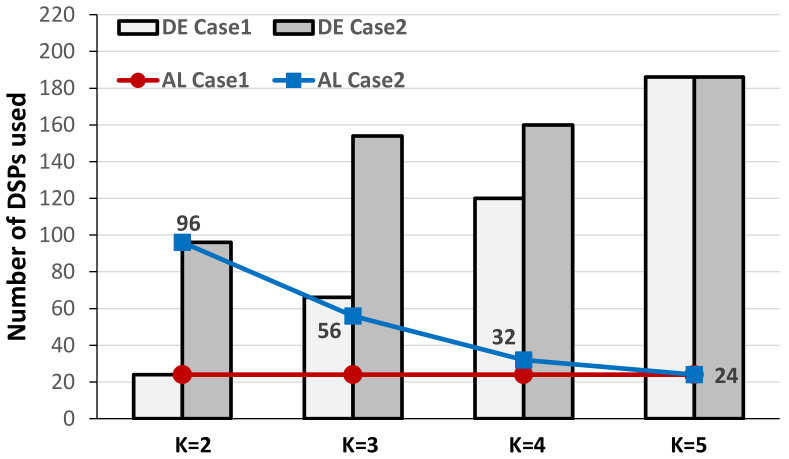
Design space exploration within the XC7Z020 device at the stride *S* = 2.

As above discussed, the computational capability actually supportable by the novel accelerator depends on the specific realization platform. In fact, it is mainly dictated by the number of required DSPs that, in turn, depends on the kernel size and the stride. However, the same number of DSPs can be exploited differently to implement different configurations of the novel DLPE, depending on the parameters *T_M_*, *T_N_*, *S*, *P_M_, and PN*. Establishing which configuration is the most appropriate for a specific operating environment is crucial to use the available resources as more efficient as possible. To this aim, different design spaces can be explored by varying the above parameters. As an example, the design space exploration reported in Figure 11 was carried out by considering the XC7Z020 device as the target, thus setting the maximum number of available DSPs to 220. The behavior of the proposed accelerator has been examined for various kernel sizes *K* and parallelism levels *T_M_* and *T_N_* with *S =* 2, *P_M_ =* 4 and *P_N_* = 1. In this condition, two different scenarios were analyzed: in the first case (the Case1 in Figure 11), *T_M_* = 2 and *T_N_* = 3 are maintained unchanged to establish the maximum supportable kernel size; conversely, in the second case (referred as the Case2) also *T_N_* varies between 24 and 6, while *T_M_* is set to 1. In the case 2, for each *K* the maximum *T_N_* has been considered (e.g., with *K* = 2, *T_N_* = 24). Figure 11 plots the numbers of DSPs used in the two referred cases by the DE and the AL versus *K*. As expected, in the first case the wider the kernel size, the higher the number of DSP slices required by the DE. On the contrary, the red line shows that the number of DSPs used to implement the fast adder trees within the AL module is maintained constant to 24, since it only depends on the parallelism and the stride. The above results show that in such a case, the maximum kernel size supportable with 220 DSPs is *K* = 5. This is the solution above referenced in Table 1 for both the SU and ES designs implemented within the XC7Z020 device. 

Results collected for the second analyzed scenario prove that to comply with the amount of DSPs on-chip available, as the kernel size increases, the parallelism must decline. Obviously, as clearly shown by the blue line in Figure 11, the lower the parallelism, the lower the number of DSPs used for accumulations. The possibility of having different design spaces to explore helps the designer in identifying the best configuration of the proposed DLPE for a certain specific operating condition. 

Finally, referring to the XC7Z020 device, the execution time of the ES implementation here proposed has been compared to a pure software design run by the 666 MHz ARM-Cortex Processor on-chip available. When executing the most complex deconvolution layer involved in the selected DCGAN model [21], the ES, which integrates the novel DLPE as the hardware accelerator, is more than 1000 times faster than the all-software implementation. 

## 5. Conclusions

This paper presented a novel hardware architecture to accelerate 2D deconvolutions in deep-learning applications. The proposed design introduced several architectural-level innovations to exploit resources available within the chosen implementation platform more efficiently than prior accelerators known in the literature. The architecture here presented has been purpose-designed to comply with the AXI4 communication protocol. Therefore, it can be integrated within virtually any heterogeneous FPGA-based SoC. The presented design is easily scalable and implementable within both high- and low-end devices, thus becoming suitable also for the integration within resource- and power-constrained embedded systems. Several implementations of both SUs and embedded systems have been characterized using different devices and parallelism levels. As a case study, the proposed architecture has been used to accelerate an existing DCGAN model. Comparisons with state-of-the-art counterparts have clearly shown the efficiency of the implementations here presented in terms of power consumption, resources requirements, and computational speed. 

## Figures and Tables

**Table 1:** Comparison results.

	Device/(Design, Precision)	*T_M_, T_N_*	LUTs	FFs	BRAMs [Mb]	DSPs	Freq. [MHz]	GOPs	Dyn. Power [Watt]
		*P_M_, P_N_*							
		*K, S*							
New	XC7Z020 (SU, 16b fix-p)	2, 3	2.9k (5.5%)	4.3k (4.1%)	0.84 (17.1%)	210 (95.5%)	200	72	0.42
		4, 1							
		5, 2							
New	XC7Z045 (SU, 16b fix-p)	2, 4	6.4k (2.9%)	9.6k (2.2%)	0.84 (4.4%)	560 (62.2%)	250	240	1.14
		8, 2							
		5, 2							
New	XC7Z100 (SU, 16b fix-p)	2, 4	15.5k (5.6%)	22.9k (4.1%)	0.84 (3.2%)	1120 (55.5%)	300	576	2.62
		16, 4							
		5, 2							
New	XC7VX690T (SU, 16b fix-p)	2, 3	23.2k (5.4%)	34.4k (4%)	0.84 (1.6%)	1680 (46.7%)	320	921.6	4.1
		32, 8							
		5, 2							
[16]	XC7VX485T (SU, 32b float-p)	4, 128	142.7k (47%)	151.4k (24.9%)	9.14 (25.2%)	2560 (91.4%)	100	NA^1^	NA
		1, 1							
		5, 2							
[15]	XC7VX690T (SU, 16b fix-p)	2, 64	304.2k (70.2%)	602.7k (69.6%)	25.03 (48.4%)	2304 (64%)	200	1578	NA
		8, 1							
		3, 1							
[17]	XC7Z100 (SU, 16b fix-p)	64, 64	117.9k (42.5%)	247.2k (44.5%)	17.4 (65.5%)	1987 (98.4%)	200	NA	2.89
		1, 1							
		3, 1							
New	XC7Z020 (ES, 16b fix-p)	2, 3	12.8k (24.6%)	17.7k (17.1%)	1.49 (30.4%)	210 (95.5%)	150	54	1.73
		4, 1							
		5, 2							
New	XC7Z045 (ES, 16b fix-p)	2, 4	16.3k (7.5%)	23k (5.3%)	1.86 (9.7%)	560 (62.2%)	167	160.3	2.3
		8, 2							
		5, 2							
[9]	XC7Z020 (ES, 12b fix-p)	NA, NA	25.5k (48%)	30.9k (29%)	2.35 (48%)	220 (100%)	100	2.6	NA
		1, 1							
		NA, NA							
[11]	XC7Z045 (ES, 16b fix-p)	NA, NA	161.8k (74%)	148.6k (34%)	15.3 (80%)	810 (90%)	150	NA	NA
		1, 1							
		5, 2							
[18]	XC7Z045 (ES, 16b fix-p)	2, 2	196.7k (90%)	NA	10.9 (57%)	603 (67%)	167	133.8	5.8
		4, 4							
		5, 2							

^1^ NA = Not Available
